# Editorial: Insights into synthetic biology 2021: Novel developments, current challenges, and future perspectives

**DOI:** 10.3389/fbioe.2023.1200227

**Published:** 2023-04-13

**Authors:** Jean Marie François, Shota Atsumi

**Affiliations:** ^1^ Toulouse Biotechnology Institute, UMR-INSA—CNRS-INRA, Toulouse, France; ^2^ Toulouse White Biotechnology Excellent Center, UMS-INSA-INRA-CNRS, Toulouse, France; ^3^ Department of Chemistry, University of California, Davis, Davis, CA, United States

**Keywords:** synthetic biology, genetic circuits, modelling, synthetic cells, metabolic engineering, sustainability, bioproduction

This “I*nsights into Synthetic Biology 2021*” Research Topic aims to illustrate the remarkable progress of synthetic biology in many different research and application areas. These include the conceptual process of building synthetic cells from scratch, sustainable solutions to our fossil dependency that can now solve greenhouse gas emissions, proposing innovative therapeutic solutions to complex diseases, or fighting infectious diseases. SB’s strength also depends on advances in methods, techniques, and algorithms, which are also presented in this Research Topic. Finally, SB is positioned in the field of education as a subject that motivates entrepreneurial aspirations.

The Research Topic “*Insight into Synthetic Biology 2021*”has been very satisfactory in providing 15 contributions, divided into 9 mini-reviews, four original papers, and two opinion papers, which together cover the current developments and challenges in Synthetic Biology (SB). Four articles were devoted to updating tools and methods for better insertion of novel functions or better control of functions in a biological system. Li et al. elegantly summarized the main pitfalls of CRISPR technology in genome editing and proposed some solutions to overcome these difficulties. Liao et al. demonstrated high antibiotic-free plasmid stability upon insertion of the *hok/sok* gene system into this plasmid, opening up the possibility of using it in large-scale process production. Regarding the methods required to implement and control new functions in biological systems, Abraha and Marchisio reported the implementation in yeast of a bacterial ClpXP protein degradation system and showed that SBML level 3 is perfectly suited to describe the modular function of this orthogonal synthetic gene circuit constructed in yeast. At a higher level of complexity are the systemic principles of gene regulatory networks, whose understanding in terms of architecture, organization, dynamics, and evolution is important for the optimal engineering of biological systems, as summarized in the study by Freyre-González et al. These authors coined the term “concilion,” which corresponds to a group of structural genes with their local regulators dedicated to a single well-defined function, as opposed to regulons or modulons, which are under the control of a specific or global regulator and that can be controlled by a diversity of functions. A biological example illustrating the concept of a concilion is provided by the response of bacteria or yeast to multi-stress, as this concilion will include several regulons organized in a regulatory cascade, each under the control of a specific regulator.

As SB is an engineering science several articles in this Research Topic are devoted to applications in different fields of biotechnology. In the field of industrial biotechnology, SB proposes sustainable alternatives aimed at reducing our dependence on fossil resources through a radical rewiring of microbial metabolism for the scalable production of a wide range of drop-in alternatives with carbon neutrality and a circular bioeconomy as the ultimate goals. Of particular relevance is the growing interest in identifying and engineering microbial systems capable of generating valuable chemicals from C1-carbon (CO_2_, methanol, and methane), thereby reducing the use of sugar feedstock and capturing atmospheric CO_2_, both of which, according to Carruthers and Lee, can transform microbial bioproduction into a more techno-economically sustainable industrial biotechnology. However, as pointed out by Treece et al., cyanobacteria would be remarkable cell factories producing chemicals from CO_2_ and sunlight if major obstacles due to low RuBisCO-dependent carbon fixation efficiency and poor growth could be overcome, which could be accomplished in the near future with the tools of SB combined with the isolation of faster-growing cyanobacteria. In support of this claim, Ferreira et al. reported the metabolic engineering of the cyanobacterium *Synechocystis* sp. PCC 6803 for glycine-betaine, showing that the production of this compatible soluble comes mostly at the expense of glycogen degradation, suggesting fixed carbon and/or energy were likely the limiting factors for this production. Synthetic methylotrophy, which is the introduction of a non-native methanol utilization pathway into a model host microorganism, is another promising topic for sustainable chemical bioproduction that also faces several scientific and technological difficulties, including the inability to grow on methanol as the sole carbon source and the very high toxicity of the intermediate formaldehyde during methanol assimilation. A solution proposed by Peiro et al. which exists in naturally methylotrophic organisms, would be to isolate the initial metabolic methanol assimilation in a compartment from its central metabolic network. SB is also relevant in many other applications like food, feed, and medicine. This is illustrated in this Research Topic by two interesting papers. The first one, presented by Cruz et al. deals with the concept of bioengineered probiotics endowed with new functionalities able to control and/or kill foodborne pathogens, induce immunity against pathogens, and eventually neutralize pathogen toxins. While there may be several benefits of this live cell biotherapeutic, including high specificity to the target, self-limiting due to lack of selection, and cost-effectiveness, several technical and ethical barriers still need to be overcome: competing with resident microbes in the gut, the induction of pathogen resistance, and biocontainment. Bioengineered probiotics have also been proposed as a next-generation product for the treatment of inflammatory bowel disease (IBD). IBD is a complex, chronic inflammatory bowel disease that primarily includes Crohn’s disease and ulcerative colitis. Although very complex, it is generally accepted that there is an association between intestinal microbiota-derived metabolites and IBD, which raises several possible clinical interventions that include delivery of soluble effector proteins inhibiting inflammatory effector genes by engineered microbes, fecal microbial transplantation, and bacteriophage therapy. As interesting and attractive as these possibilities are, the main obstacle remains the safety of genetically modified organisms in these innovative therapeutic approaches.

Finally, the demonstration that SB is an elective field in science is illustrated on the one hand by the article by Stano (2022), which presents his opinion on the design, function, and application of artificial cellular systems, and by the report of an iGEM-inspired applied program given to undergraduate students, showing improved skills, talent, and entrepreneurial motivation (Gill et al., 2022).
